# A Facile Method to Fabricate an Enclosed Paper-Based Analytical Device via Double-Sided Patterning for Ionic Contaminant Detection

**DOI:** 10.3390/bios13100915

**Published:** 2023-10-05

**Authors:** Jinsol Choi, Eun-Ho Lee, Sung-Min Kang, Heon-Ho Jeong

**Affiliations:** 1 Department of Chemical and Biomolecular Engineering, Chonnam National University, 50 Daehak-ro, Yeosu 59626, Jeollanam-do, Republic of Korea; cjs0503@jnu.ac.kr; 2 Department of Green Chemical Engineering, Sangmyung University, 31 Sangmyungdae-gil, Cheonan 31066, Chungcheongnam-do, Republic of Korea; 202121225@sangmyung.kr; 3 Future Environment and Energy Research Institute, Sangmyung University, 31 Sangmyungdae-gil, Cheonan 31066, Chungcheongnam-do, Republic of Korea

**Keywords:** microfluidic paper-based analytical device, double-sided patterning, enclosed channel, ionic contaminant, multiplex detection

## Abstract

Microfluidic paper-based analytical devices (μPADs) have been developed for use in a variety of diagnosis and analysis fields. However, conventional μPADs with an open-channel system have limitations for application as analytical platforms mainly because of the evaporation and contamination of the sample solution. This study demonstrates the design and fabrication of an enclosed three-dimensional(3D)-μPAD and its application as a primary early analysis platform for ionic contaminants. To generate the hydrophobic PDMS barrier, double-sided patterning is carried out using a PDMS blade-coated stamp mold that is fabricated using 3D printing. The selective PDMS patterning can be achieved with controlled PDMS permeation of the cellulose substrate using 3D-designed stamp molds. We find the optimal conditions enabling the formation of enclosed channels, including round shape pattern and inter-pattern distance of 10 mm of stamp design, contact time of 0.5 min, and spacer height of 300 µm of double-sided patterning procedure. As a proof of concept, this enclosed 3D-μPAD is used for the simultaneous colorimetric detection of heavy metal ions in a concentration range of 0.1–2000 ppm, including nickel (Ni^2+^), copper (Cu^2+^), mercury (Hg^2+^), and radioactive isotope cesium-137 ions (Cs^+^). We confirm that qualitative analysis and image-based quantitative analysis with high reliability are possible through rapid color changes within 3 min. The limits of detection (LOD) for 0.55 ppm of Ni^2+^, 5.05 ppm of Cu^2+^, 0.188 ppm of Hg^2+^, and 0.016 ppm of Cs+ are observed, respectively. In addition, we confirm that the analysis is highly reliable in a wide range of ion concentrations with CV values below 3% for Ni^2+^ (0.56%), Cu^2+^ (0.45%), Hg^2+^ (1.35%), and Cs^+^ (2.18%). This method could be a promising technique to develop a 3D-μPAD with various applications as a primary early analysis device in the environmental and biological industries.

## 1. Introduction

Water pollution by ionic contaminants is a serious global issue caused by exposure to environmental pollutants such as strong acids/bases, heavy metals, and radioactive isotopes [1,2,3,4,5]. For example, the indiscriminate discharge of manufacturing wastewater can lead to water pollution with strong acids/bases and heavy metal ions, bringing life on Earth under direct threat [2,3,6]. Furthermore, the contamination of water from radioactive isotope exposure has been a serious social problem since the Fukushima nuclear disaster in 2011, and their high-water solubility and extended half-life can lead to their accumulation in vivo, inducing gene mutations and cancer cell expression [7,8]. Therefore, the development of screening platforms for the easy detection and quantification of ionic contaminants is challenging. Various techniques, including atomic absorption [9,10], optical chemical sensors [11,12], instrumental neutron activation analysis [13,14], electrochemical sensors [15,16], inductively coupled plasma-mass spectrometry (ICP-MS) [17,18], and X-ray fluorescence spectroscopy [19,20], have been reported for water pollution analysis owing to their high accuracy and selectivity [21,22]. However, these techniques are costly, time-consuming, and require sophisticated sample preparation, experienced specialists, and inactivation of samples for in situ monitoring in the real field [23]. Thus, before undertaking analysis using high-performance equipment, it is important to establish a primary early detection technique for the simple on-site analysis of target samples.

Recently, microfluidic paper-based analytical devices (μPADs) have emerged as promising assay platforms for cost-effective on-site testing with a low sample volume and end-user convenience [24,25,26]. For the fabrication of PADs, various techniques have been explored, including photolithography [27], wax printing [28], ultraviolet/ozone (UV/O_3_) patterning [29], and three-dimensional (3D) printing [30]. Common two-dimensional (2D) open-channel μPAD test designs enable control of the lateral fluid flow over the hydrophilic paper channel, which is defined by the geometry of the hydrophobic barrier. Such a device has been applied to various fields, especially in environmental science [23,31,32] and clinical diagnosis [33,34,35,36,37]. However, because both sides of the basic 2D-μPADs are uncovered, they have the intrinsic limitation of sample evaporation, resulting in low detection efficiency and accuracy due to the very low sample volume used, in addition to the risk of sample contamination [23,38].

To address the issues of the basic 2D open-channel design, several methods for sealing one or both sides of the 3D-μPADs have been explored. Attempts at one-side sealing have been made to form a 3D channel for improving detection sensitivity using accurate flow control [37,38,39,40]. Fabrication of these 3D-μPADs can be achieved by stacking and origami of 2D-μPADs using adhesive tape but requires a complex and specific procedure for alignment and complete bonding. More favorable fabrication strategies for 3D-μPADs are automatic printing approaches, including wax printing and 3D printing [41,42]. Despite the easy fabrication of these 3D-μPADs, the open channel means that evaporation and contamination of the sample solution can still occur. Therefore, fully enclosed 3D-μPADs, which have both sides sealed, have been proposed [43,44]. With wax printing, the formation of a 3D hydrophobic wax barrier on the paper is achieved by controlling the lateral and vertical penetration of the melted wax pattern on both sides of the paper. Although wax printing is beneficial for the fabrication of enclosed channels with high resolution in a single sheet of paper, this technique has limitations such as complicated alignment of the printed wax pattern and the incompatibility of the wax material with some chemicals [45]. Alternatively, poly(dimethylsiloxane) (PDMS) has become a very valuable material as a hydrophobic barrier coated on paper, owing to its transparency, low cost, convenient use, and compatibility with some organic chemicals [46]. The fabrication methods for PDMS-based μPADs have been reported in previous studies including the use of rubber stamps for contact printing and stencil mesh for screen printing [47,48]. The micro-patterned stamp and stencil screen with patterned mesh holes are used to selectively transfer PDMS ink to the paper surface. Designed PDMS hydrophobic barriers are formed by penetrating the PDMS into pores of paper and then thermal curing. However, an enclosed 3D-μPAD fabricated using PDMS has rarely been reported.

In this study, we present a facile method for the fabrication of an enclosed PAD with 3D PDMS barriers, as well as its potential application in the detection of ionic pollutants, including heavy metals and radioactive isotopes. The enclosed channels are formed by controlling the lateral and vertical penetration of PDMS that is selectively loaded on both sides of the paper using a double-sided contact printing method. We accomplish this using a 3D-printed stamp mold with a blade-coated PDMS thin film. We identify that the determination of the enclosed channel formation is dependent on the negative pattern design of the stamp mold, such as the ratio of width to inter-channel distance, inter-pattern spacing, shape, and depth. In addition, the enclosed hydrophilic channel is affected by the double-sided printing conditions, which include the contact time between the stamp and paper, and the thickness of the PDMS coated onto the stamp. To demonstrate the versatility of the enclosed 3D-μPAD, we introduce a colorimetric reagent capable of detecting heavy metal ions such as copper (Cu^2+^), nickel (Ni^2+^), and mercury (Hg^2+^), as well as radioactive cesium-137 ions (Cs^+^). These ionic contaminants in water are detected by identifying the colorimetric change in an unknown sample within 3 min and are quantified by measuring the color intensity. Thus, the enclosed 3D-μPAD developed in this study can be used as a primary early detection sensor and contributes to the development of the fields of environmental purification and biosensors by enabling multiple analyses of unknown samples.

## 2. Materials and Methods

### 2.1. Materials

The Sylgard 184 and curing agent were purchased from Dow Corning (Midland, MI, USA). Copper(II) sulfate pentahydrate (CuSO_4_∙5H_2_O, ≥98%), nickel(II) sulfate hexahydrate (NiSO_4_∙6H_2_O, ≥98%), mercury(II) chloride (HgCl_2_, ≥99.5%), and cesium chloride (CsCl, ≥99.999%) were purchased from Sigma-Aldrich (St. Louis, MO, USA). The reagents for the detection of each element, namely potassium iodide (KI, ≥99.99%), dimethylglyoxime (DMG, ≥99%), 4,4′-bis(dimethylamino)thiobenzophenone (Michler’s thioketone, MTK, ≥85%), and 4-phenylazo-*m*-phenylenediamine monohydrochloride (chrysoidine G, CG, ≥95%) were purchased from Sigma-Aldrich.

### 2.2. Fabrication of Stamp Molds

The blade coating of the stamp mold was used to create selective patterning on hydrophilic cellulose chromatography paper (Whatman 3MM chromatography paper, Whatman International Ltd., Maidstone, UK). The stamp molds, including round, rectangular, and trapezoidal shapes, were designed using 3D CAD software (Inventor 2022, Autodesk Inventor Fusion, Autodesk Inc., San Rafael, CA, USA), then translated to an STL file format, and fabricated using a high-resolution PolyJet technique 3D printer (Objet30 3D printer, Stratasys Ltd., Eden Prairie, MN, USA). The printed stamp mold was immersed into a solution of sodium metasilicate anhydrous (Na_2_SiO_3_, ≥95%) and sodium hydroxide (NaOH) beads (20–40 mesh, 97%) mixed in distilled water in a 1:2 ratio and sonicated for 2 h to remove the support materials. Then, the stamp mold was washed three times with ethanol and deionized water repeatedly and dried at room temperature.

### 2.3. Fabrication of Enclosed 3D-μPAD

**Figure 1:**
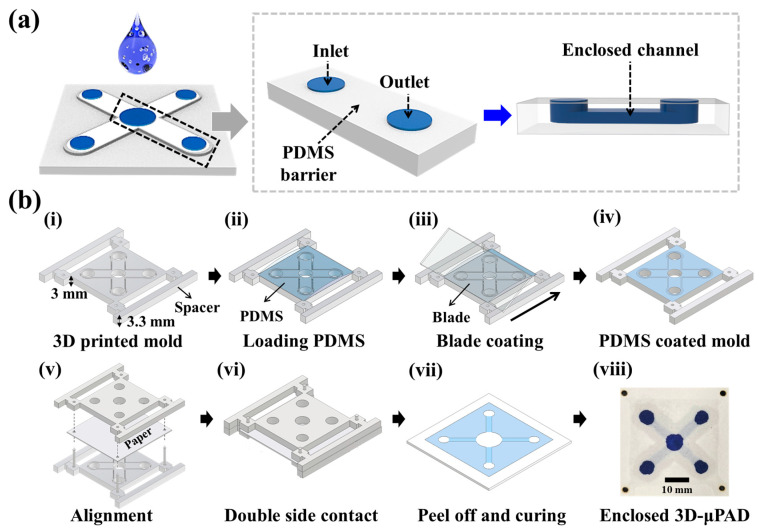
Design and fabrication procedure for enclosed 3D-PAD. (**a**) Schematic illustration of enclosed 3D-PAD with four channels for multiplex detection. (**b**) Schematic illustration of stepwise procedures to form hydrophobic PDMS barriers using blade coating and double-sided patterning. (i) 3D printing of stamp mold. (ii) loading of PDMS mixture solution into mold. (iii,iv) Formation of thin film of PDMS via blade coating. (v) alignment of paper and PDMS coated mold using four alignment pins. (vi) The PDMS solution is immediately transferred to the paper via double-sided contact printing. (vii) peeling off the stamp molds and then thermal curing of PDMS. (viii) Optical image for enclosed 3D-μPAD.

To form this enclosed 3D-μPAD, we performed a double-sided printing process for patterning the paper, as described in our previous report [23]. First, PDMS was mixed in the ratio of 10:1 with Sylgard 184 precursor and curing agent, and degassing procedures were performed in a vacuum chamber until no bubbles were observed. We prepared the stamp mold using a 3D printer (Figure 1b(i)) and approximately 1.0 mL of uncured PDMS mixture was loaded onto the 3D printed stamp mold using a pipette (Figure 1b(ii)). Then, a thin layer of PDMS was formed by removing excess PDMS using the blade (Figure 1b(iii,iv)). For the double-sided patterning, two stamp molds were aligned with the paper by using four stainless steel pins and then put in contact to induce PDMS permeation of the paper (see Figure 1b(v–vii)). Holes in the paper for connecting the alignment pins were formed with a laser machine (NOVA24, Thunder Laser Tech Co., Ltd., Shatian, China). After that, the disassembled paper underwent thermal polymerization of PDMS for 3 min on a hot plate at 100 °C.

### 2.4. Colorimetric Detection of Heavy Metal Ions and Radioactive Isotope

The colorimetric detection sites were formed using the drop-casting method [12,49]. Briefly, 3 µL of the colorimetric agents, including DMG (10 mg/mL) in ethanol, KI (0.4 M) in acetone, MTK (10 mg/mL) in acetone, and CG (0.25 mg/mL) in distilled water, respectively, was loaded into each detection site and then allowed to dry in an oven at 95 °C for 30 min. The artificially contaminated water sample containing Cu^2+^, Ni^2+^, Hg^2+^, and Cs^+^ at various concentrations (100, 250, 500, 1000, and 2000 ppm) was loaded into the center site, and then the colorimetric change was monitored with the naked eye.

### 2.5. Analysis Method

The formation of the enclosed channel was characterized by adding 1 wt% methylene blue (MB) dye in distilled water to the loading site. All optical images were obtained using a stereo zoom microscope (SM-4TZZ-144A, AmScope, Irvine, CA, USA). Quantitative analysis was conducted using a smartphone camera in high dynamic range (HDR) mode. The color changes of each detection site corresponding to Ni^2+^, Cu^2+^, Hg^2+^, and Cs^+^ were recorded using a desktop scanner and converted into image data in TIF format. The numerical colorimetric change values of the images were extracted from nine-grid points using ImageJ software (NIH, Bethesda, MD, USA) [50,51]. The red, green, and blue (RGB) values of the before and after sensing images were used to calculate the ΔR, ΔG, and ΔB values, and the normalized intensity was calculated using the following equation: [52]

(1) $$\mathrm{Normalized}\;\mathrm{intensity}\;\left(\Delta R,\;\Delta G,\;\Delta B\right)=\frac{\mathrm{Target}\;\mathrm{color}\;\mathrm{value}}{R\;\mathrm{value}+G\;\mathrm{value}+B\;\mathrm{value}}\;$$

where the R, G, and B values are the numerical single-color coordinate values of the colorimetric images before sensing, respectively.

In addition, the limit of detection (LOD) was obtained from the plot of the total RGB value versus concentration of each ionic sample (in ppm) and calculated using the Equation (2):

(2) $$\mathrm{limit}\;\mathrm{of}\;\mathrm{detection}\;\left(\mathrm{LOD}\right)=\frac{3S_{b}}{S}\;$$

where *S_b_* and *S* are the standard deviation of the blank and the slope of the total RGB value after sensing of Ni^2+^, Cu^2+^, Hg^2+^, and Cs^+^, respectively.

## 3. Results

### 3.1. Fabrication of Enclosed 3D-μPAD

As illustrated in Figure 1a, we designed the enclosed 3D-μPAD with four multiplex channels for multiplex detection of ionic contaminants, including Ni^2+^, Cu^2+^, Hg^2+^, and Cs^+^. The 3D-μPAD consists of one sample loading site and four detection sites. These sites are interconnected by underpass channels in the paper. To fabricate the enclosed 3D-μPAD, blade coating and double-sided patterning methods are applied to form the PDMS hydrophobic barrier using a 3D-printed stamp mold. The design of the stamp mold is critical to form an enclosed channel, which consists of a desired negative pattern, holes for channels and alignment, and spacers for uniform coating (see Figure 1b(i)). During the blade coating process, the spacer contributes to guiding the PDMS coating with uniform thickness by removing residual PDMS using the blade (see Figure 1b(ii–iv)). After blade coating, no PDMS coating occurs on holes and relatively thick PDMS layers are formed onto negative patterns. To conduct double-sided patterning of PDMS, alignment between the PDMS-coated stamp and paper is a critical step. Alignment pins connect to holes designed in both the stamp mold and paper, and they can be fixed strongly (see Figure 1b(v)). After fitting and contacting the stamp mold to the top and bottom sides of the paper, liquid PDMS penetrates the paper differentially as the thickness of the PDMS layer is determined by the negative pattern of the stamp (see Figure 1b(vi)). Finally, hydrophobic PDMS barriers are formed after peel-off and thermal curing (see Figure 1b(vii)). We confirm that enclosed channels are formed in paper by identifying different color intensities of dye between the sample loading sites and hydrophilic channel sites when dye solution (i.e., MB) is introduced to the center site of the μPAD (see Figure 1b(viii)).

### 3.2. Effect of Stamp Design on PDMS Penetration of Paper

**Figure 2:**
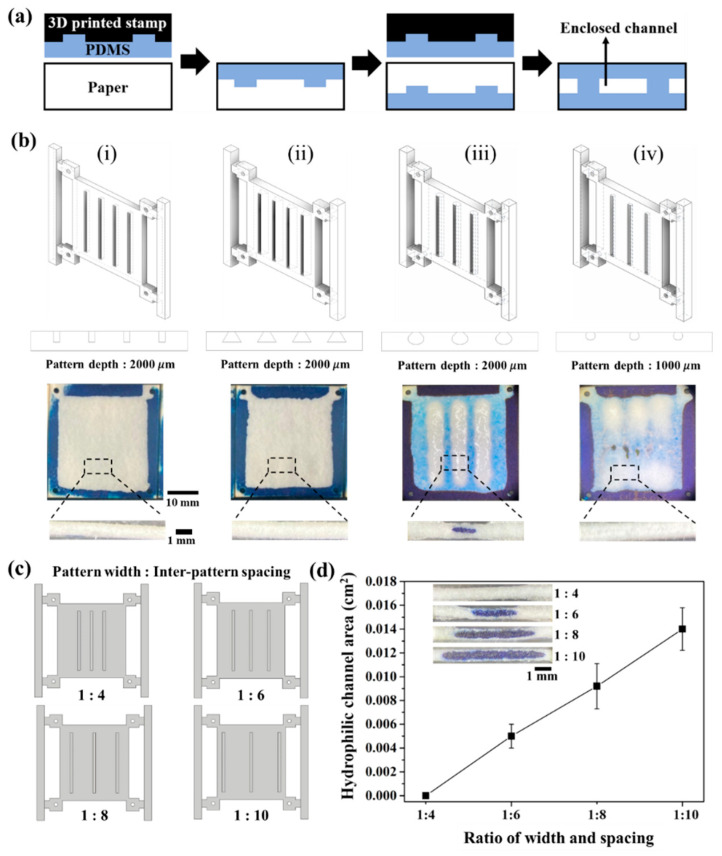
Effect of stamp mold geometry on enclosed channel formation in paper. (**a**) Schematic illustration of double-sided imprinting for the generation of hydrophobic barriers. (**b**) Schematic illustration of various shapes of the stamp mold including (i) rectangular, (ii) trapezoidal, (iii) round with low depth, and (iv) round with high depth and its optical images for patterned PDMS barriers. (**c**) Schematic illustration of changes in the different ratios (channel width: inter-channel distance) of PDMS-coated mold. (**d**) The formation of a hydrophilic channel at various width ratios. The inset in (**c**) shows the cross-section images of the generated hydrophobic barrier by flowing methylene blue (MB) aqueous solution on the hydrophilic channel. The results represent the mean (±SD) of ten independent experiments.

The double-sided patterning of PDMS can form 3D hydrophobic barriers that serve as the boundary of the enclosed hydrophilic channels, allowing directional fluid flow without evaporation. In this method, the design of the stamp mold plays a critical role in the formation of the enclosed channel. Our strategy to fabricate the enclosed channel is the differential penetration of PDMS into the paper by varying the volume of PDMS coated on the stamp mold (Figure 2a). The volume of PDMS to be loaded onto paper is determined using the void of the negative pattern. A higher volume of PDMS can lead to more penetration into the paper laterally and vertically. With double-sided patterning, the PDMS liquid, showing a greater degree of penetration on both sides of the paper, merges and forms complete hydrophobic barriers. Meanwhile, a relatively low volume of PDMS penetrates into only part of the paper and does not merge with the penetrated PDMS on the other side, resulting in the formation of an enclosed channel.

First, we investigated the effect of the geometry of the stamp mold on the formation of the enclosed channel, including round, rectangular, and trapezoidal shapes (Figure 2b). We used a PolyJet 3D printer to print stamp molds with 3D geometries, which is difficult to achieve with conventional lithography, and we tested the possibility of enclosed channel formation. The results demonstrate that the enclosed channels are well-formed using the round geometry of the stamp pattern compared to rectangular and trapezoidal patterns (Figure 2b(i–iii)). In addition, no enclosed channel is formed when the round pattern has low depth (Figure 2b(iv)). The results clearly show that for a given round shape of the stamp pattern, the amount of PDMS stored within the void pattern is closely related to the geometry of the stamp pattern, which affects PDMS penetration into the paper.

Next, we investigated the effect of inter-pattern spacing on the enclosed channel of paper using a round-shaped stamp. We tried to form the enclosed channel using stamp molds with different ratios of pattern depth (2000 μm) to spacing (4, 6, 8, and 10 mm), ranging from 1:4 to 1:10 (Figure 2c), and measured the dyed area of the enclosed channel. As shown in Figure 2d, the areas of enclosed channels are varied by changing the distance between round patterns. The hydrophilic channels are not formed using a 1:4 ratio of stamp mold, and the area of the enclosed channel formed increases as the inter-pattern distance increases. These results indicate that the short inter-channel distance induces the merging of vertically penetrated PDMS liquids during the double-sided contact time (30 s), resulting in channel clogging. Sufficient distance to prevent merging between laterally penetrating PDMS liquids is achieved at ratios above 1:6.

### 3.3. Controlled Formation of Enclosed Channel Using Double-Sided Patterning

**Figure 3:**
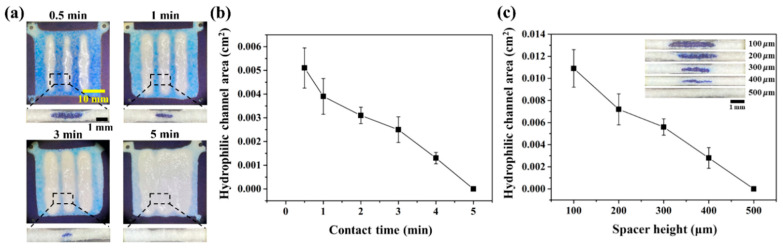
Controlled formation of enclosed channel by varying contact time and spacer height. (**a**) Optical images for formation of enclosed hydrophilic channels with contact time. (**b**) Changes in hydrophilic channel area with increasing contact time from 0.5 to 5 min. (**c**) Changes in hydrophilic channel area with increasing the spacer height of stamp mold from 100 to 500 μm and its corresponding inset cross-sectional images. Error bars are smaller than symbols for data points at 5 min of contact time and 500 μm of spacer height. The results represent the mean (±SD) of ten independent experiments.

To demonstrate the feasibility of controlled formation of enclosed channels using double-sided patterning, we varied the contact time and spacer height while maintaining the use of a stamp mold with a round pattern (2000 μm depth and 1:6 ratio of spacing), as shown in Figure 3. According to the Lucas–Washburn equation, the spreading distance by capillary flow in porous media is dependent on the viscosity of PDMS liquid, surface tension, average pore diameter, and time [53,54,55]. The contact time between the PDMS-coated stamp mold and papers determines the PDMS penetration time into paper. As shown in Figure 3a, optical images of the top and side views show the change in hydrophobic PDMS barrier (white region) with increasing contact time from 0.5 to 5 min. We quantify the cross-section area of the enclosed channel using the side view. Figure 3b shows that the area of the hydrophilic enclosed channel decreases until 4 min, and no formation occurs at 5 min. The spacer height, which is a support that can assist uniformity of the PDMS blade coating, can be varied with the 3D model design for 3D printing. Figure 3c shows that the area of the hydrophilic enclosed channel also decreases with increasing spacer height from 100 to 400 μm, and no formation occurs at 500 μm. This result indicates that the height of the spacer is related to the initial PDMS loading volume onto the paper, implying that there is a limit to the amount of PDMS penetration for forming high-resolution and sophisticated channels.

To efficiently prevent sample evaporation, a thick channel wall is required on the top and bottom side of paper because thin walls can induce sample evaporation due to the gas permeability of PDMS. In addition, blockage of enclosed channels in all areas should be prevented to successfully implement multiplex ion detection using enclosed 3D-μPAD. For this, we applied the optimal conditions enabling stable formation of enclosed channels for the ion detection application, including inter-channel distance of 10 mm, contact time of 0.5 min, and spacer height of 300 μm. 

### 3.4. Application of Colorimetric Sensor for Simultaneous Detection of Heavy Metals and Radioactive Isotope

Previous reports using enclosed paper-based channels have shown improved detection sensitivity compared to open-channel μPADs because of no sample evaporation. We can also successfully fabricate 3D-μPADs with enclosed hydrophilic channels that can be employed as primary early warning sensors for water pollution detection.

**Figure 4:**
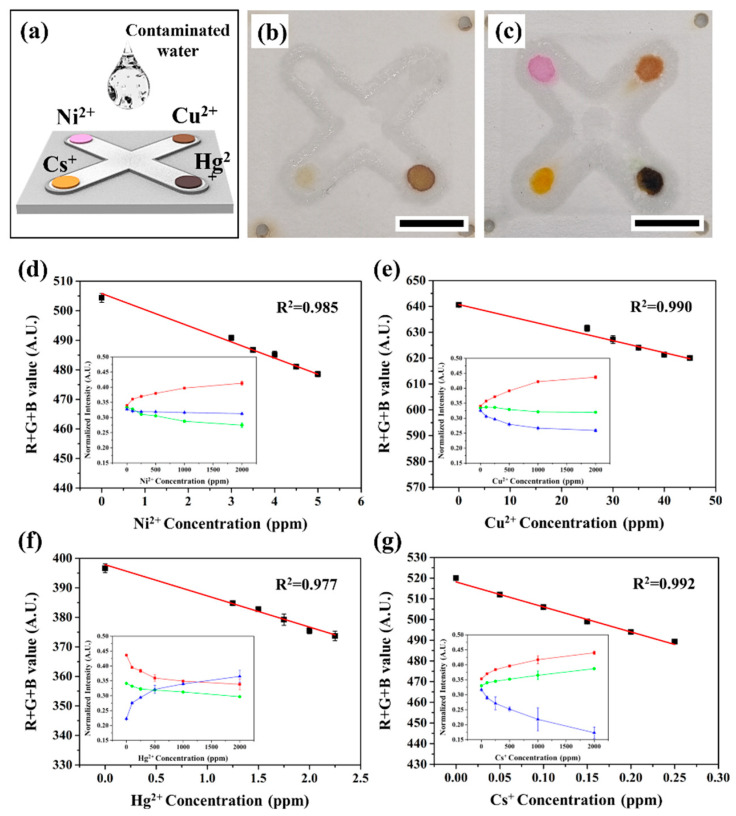
Colorimetric detection of various ionic contaminants using enclosed μPAD. (**a**) Multiplex colorimetric response for detection of water samples contaminated with nickel (Ni^2+^), copper (Cu^2+^), mercury (Hg^2+^), and cesium (Cs^+^) ions. Brightfield image of colorimetric detection (**b**) before and (**c**) after the addition of the contaminated water sample. Linear relationship between the gray values in the presence of (**d**) Ni^2+^, (**e**) Cu^2+^, (**f**) Hg^2+^, and (**g**) Cs^+^, covering the range from 0.1 to 50 ppm. The inset graph shows RGB colorimetric changes in normalized intensity with the different concentrations of Ni^2+^, Cu^2+^, Hg^2+^, and Cs^+^ ions, respectively. The lines in red, green and blue indicate R-, G-, and B- intensity values. Error bars are smaller than symbols for all data points (n = 6 individual closed circles). T = 298 K, pH = 6.0 ± 0.1, and t_eq_ = 3 min. The results represent the mean (±SD) of ten independent experiments.

Figure 4 shows the paper-based colorimetric sensing for primary early detection of heavy metal ions (Cu^2+^, Ni^2+^, and Hg^2+^) and a radioactive isotope (Cs^+^) in a water environment, as well as a cross-shaped enclosed hydrophilic channel to conduct simultaneous detection of multiple contaminants in a single 3D-μPAD. The μPAD consists of four detecting zones (i.e., edge arms) impregnating different chemosensors, and one sample loading zone (i.e., center), resulting in the colorimetric reaction at the end of the channels for each element (Figure 4a,b). After loading the simulated water pollutant containing heavy metal and radioactive isotope ions, colorimetric changes (pink: Ni^2+^ ions, yellow: Cu^2+^ ions, dark brown: Hg^2+^ ions, red: Cs^+^ ions, respectively) occur within a few minutes (<3 min), as illustrated in Figure 4c.

This result demonstrates that the ion-contaminated aqueous solution is delivered to the detecting zones by capillary force in the enclosed hydrophilic channel that can avoid sample loss and contamination because of the hydrophobic PDMS barrier. By discriminating the color changes of the μPAD in accordance with the interaction of the chemosensor with each contaminant, we collected the image data using a smartphone camera and demonstrated the ability to quantify the sample solution through the conversion of mathematical RGB color values for R, G, and B. The intensity change is clearly observed with increasing the initial concentration of each ion; the concentration is varied from 0 to 2000 ppm (insert Figure 4d–g). In detail, the R-value gradually increases with the concentration of Ni^2+^, and the G- and B-values decrease (insert Figure 4d). In the case of Cu^2+^, the R-value increases, whereas the B-value decreases significantly (insert Figure 4e). In addition, in the case of Hg^2+^, the R- and G-values decrease, whereas the B-value increases as the concentration increases (insert Figure 4f). Finally, the colorimetric response of Cs^+^ shows that the R- and G-values increase with increasing concentration of Cs^+^, and the B-value decreases (insert Figure 4f). The enclosed 3D-μPADs show good detection sensitivity in the range from 0.1 to 50 ppm compared to previous research [12,31,56,57], and provide a limit of detection (LOD) of 0.55 ppm of Ni^2+^, 5.05 ppm of Cu^2+^, 0.188 ppm of Hg^2+^, and 0.016 ppm of Cs^+^, respectively (Figure 4d–g). In addition to quantitative analysis, we measure the coefficient of variation (CV) values from ten independent enclosed 3D-μPADs to identify statistically reliable analysis (CV < 5%). We confirm that highly reliable analysis with CV values below 3% for Ni^2+^, Cu^2+^, Hg^2+^, and Cs^+^ in a wide range of ion concentrations is achieved using enclosed 3D-μPADs. These results suggest that the enclosed multi-channel μPAD developed in this study can potentially be used for the qualitative and quantitative analysis of various ionic contaminants, and the hydrophilic channels flanked with hydrophobic barriers prevent sample loss and leakage, providing a useful early detection platform for end users. Furthermore, our platform has invaluable practical potential for on-site environmental detection and real-time monitoring.

## 4. Conclusions

In this paper, we describe the development of a 3D-μPAD composed of enclosed channels, using PDMS blade coating and a double-sided patterning technique, for the rapid detection of ionic contaminants. We find the optimal parameters for the controlled formation of complete enclosed channels, including an inter-channel distance of 10 mm, a contact time of 0.5 min, and a spacer height of 300 µm. Using this 3D-μPAD, we demonstrate the highly reliable detection (CV < 3%) of heavy metal ions Ni^2+^, Cu^2+^, and Hg^2+^, and a radioactive isotope ion Cs^+^ by monitoring the colorimetric change in sensing probes using a smartphone camera. The detection procedure from sample loading to color change is completed within 3 min. Therefore, it is anticipated that the enclosed 3D-μPAD could be useful in primary early detection with numerous applications as an analytical platform with high efficiency and versatility, particularly in the fields of environmental pollutant detection and biomedical diagnosis.

## Data Availability

Not applicable.
